# Pregnancy and related complications in achondroplasia: a scoping review

**DOI:** 10.1186/s12884-026-09570-8

**Published:** 2026-07-01

**Authors:** Krupa Shah, Kosha Sheth, Dhairya Shah, Hitesh Shah

**Affiliations:** 1 Department of Obstetrics and Gynaecology, Kasturba Medical College, Manipal Academy of Higher Education, Manipal, India; 2 Consultant, Apple Children Hospital, Eillisbridge, Ahmedabad India; 3 Medical Student, Father Muller Medical College, Mangalore, India; 4 Paediatric Orthopaedics Department, Kasturba Medical College, Manipal Academy of Higher Education, Manipal, Karnataka State 576 104 India

## Abstract

Very few articles have investigated pregnancy in women with achondroplasia. A scoping review methodology was used to record and summarize the existing research evidence. The review process was conducted in accordance with the PRISMA-ScR guidelines. Original studies and case series mentioning achondroplasia with a minimum of 5 pregnancies were included. A total of 1527 articles were screened by title and abstract. Twenty-one full-text articles were reviewed for eligibility criteria. Seven studies were included. The reference details, study characteristics, topics of interest, and main findings are presented. The combination of short stature and a narrow pelvis led to a difficult antenatal and intrapartum period. Over the past decades, the number of publications on achondroplasia has increased overall, but pregnancy-related articles are still lacking. Multicentre studies should be initiated following methodological reference standards to strengthen the reliability and generalizability of the findings and to increase their relevance for implementation in clinical practice.

## Introduction

Achondroplasia is the most common form of skeletal dysplasia [1]. It is rare, with a prevalence of approximately 1 in 30,000 individuals [2]. The common clinical features include disproportionate short stature, short limbs, rhizomelia, macrocephaly, midface hypoplasia, genu varum, thoracolumbar kyphosis, and lumbar canal stenosis. Spinal canal stenosis with neurological claudication is more common in adults. Achondroplasia is found equally in both genders. 

A woman with achondroplasia generally experiences normal puberty and menarche; however, fibroids, endometrial polyps, and ovarian cysts are common gynecological issues [3]. There are unique obstetric concerns among women with achondroplasia [3]. The short stature and narrow pelvis make antenatal and intrapartum periods comparatively eventful. These women may face many barriers related to obstetric and gynecological health care during their lifetime [4–6].

A greater age of pregnancy reflected reduced reproductive fitness, leading to subfertility and late socialization in the opposite sex. The obstetric journey starts with marriage or having a partner to a woman with achondroplasia. The likelihood of having a child with a similar disorder and burden should always be weighed against the happiness of the couple. Hence, genetic counseling is an essential part of the premarital and preconception periods. Accurate molecular diagnosis, in addition to clinical diagnosis, is crucial for counseling. Preconceptional and prenatal counseling is crucial for the detection of a fetus with achondroplasia and may reduce obstetric complications in pregnant women with achondroplasia.

Owing to the rarity of the combination of pregnancy and achondroplasia, the literature is limited. Pregnancies in women with achondroplasia face unique challenges during the antepartum, intrapartum, and postnatal periods. Many reports suggest delivery routes involving only c-sections for pregnant women with achondroplasia. Owing to cervical spine instability and other anatomical differences, anesthesia for achondroplasia is also challenging [7, 8]. Fewer studies exist related to pregnancy and its complications in women with achondroplasia [1–5, 9, 10]. We have not found any scoping or systematic reviews related specifically to pregnancy and achondroplasia in the literature. Hence, we decided to perform a scoping review of pregnancy and related complications in patients with achondroplasia.

### The aims of the study

The objective of this study was to systematically review the available research on pregnancies in a woman with achondroplasia, which includes antenatal, intrapartum, and postnatal fetal and maternal outcomes, via a scoping review methodology.

## Materials and methods

### Study design

The study was conducted in accordance with the PRISMA-Scr guidelines, which include research questions; identifying appropriate studies; selecting, extracting, and charting data; and collating, summarizing, and reporting the results of selected studies related to the study questions. A study protocol was developed.

Definition of the research question: The formulation of the search string followed the PCC system

- Population Pregnancies with achondroplasia
- Concepts – Feto-maternal outcomes and delivery
- Context – Studies in English

The protocol was not previously registered.

### Search strategies

A systematic literature search was carried out in PubMed, Medline, Embase, and CINAHL. The MeSH words used were “achondroplasia”, “dwarf” or “dwarfism” and “pregnancy” or “cesarean deliveries”, “anesthesia”. Achondroplasia related to humans was used. No time limits were used. Two experienced consultants approved the search strategy. The content of interest was antepartum, intrapartum, and postpartum outcomes. The topics of interest were antenatal complications, including anemia, preeclampsia, diabetes, respiratory discomfort of mothers, pregnancy loss (abortions, termination of pregnancy), mode of delivery, anesthesia and related complications, and fetal achondroplasia.

### Inclusion criteria

All original studies and surveys of pregnant women with achondroplasia and/or a case series of pregnant women with achondroplasia with a minimum of 5 pregnancies were included. Only quantitative studies were included. Only studies in the English language were included.

### Exclusion criteria

Review articles, conference proceedings, original articles without pregnancies with achondroplasia, case series with fewer than 5 pregnancies with achondroplasia, expert comments, guidelines and qualitative studies were excluded. Studies concerning only genetics, diagnostics, and prenatal diagnosis were excluded.

### Study selection, data extraction, and synthesis

Two reviewers (* and **) independently screened the title and abstract to identify potentially eligible articles. The full-text articles were selected and assessed for eligibility by two reviewers to obtain the final study articles. One reviewer (*) studied the inclusion and exclusion criteria, and one reviewer (**) extracted the data from eligible studies. The first reviewer verified the results to ensure the accuracy of the synthesis. An a priori data extraction form was used. Any disagreements in the process of study selection and data extraction were resolved by discussion with the other reviewer. An assessment of the methodology quality and bias of the included studies was not performed. The included articles were presented in texts and tables. The data extracted included antepartum complications, medical and obstetrical complications, types of delivery, and maternal and fetal outcomes.

## Results

### Search results, inclusion criteria, and exclusion criteria

**Fig. 1:**
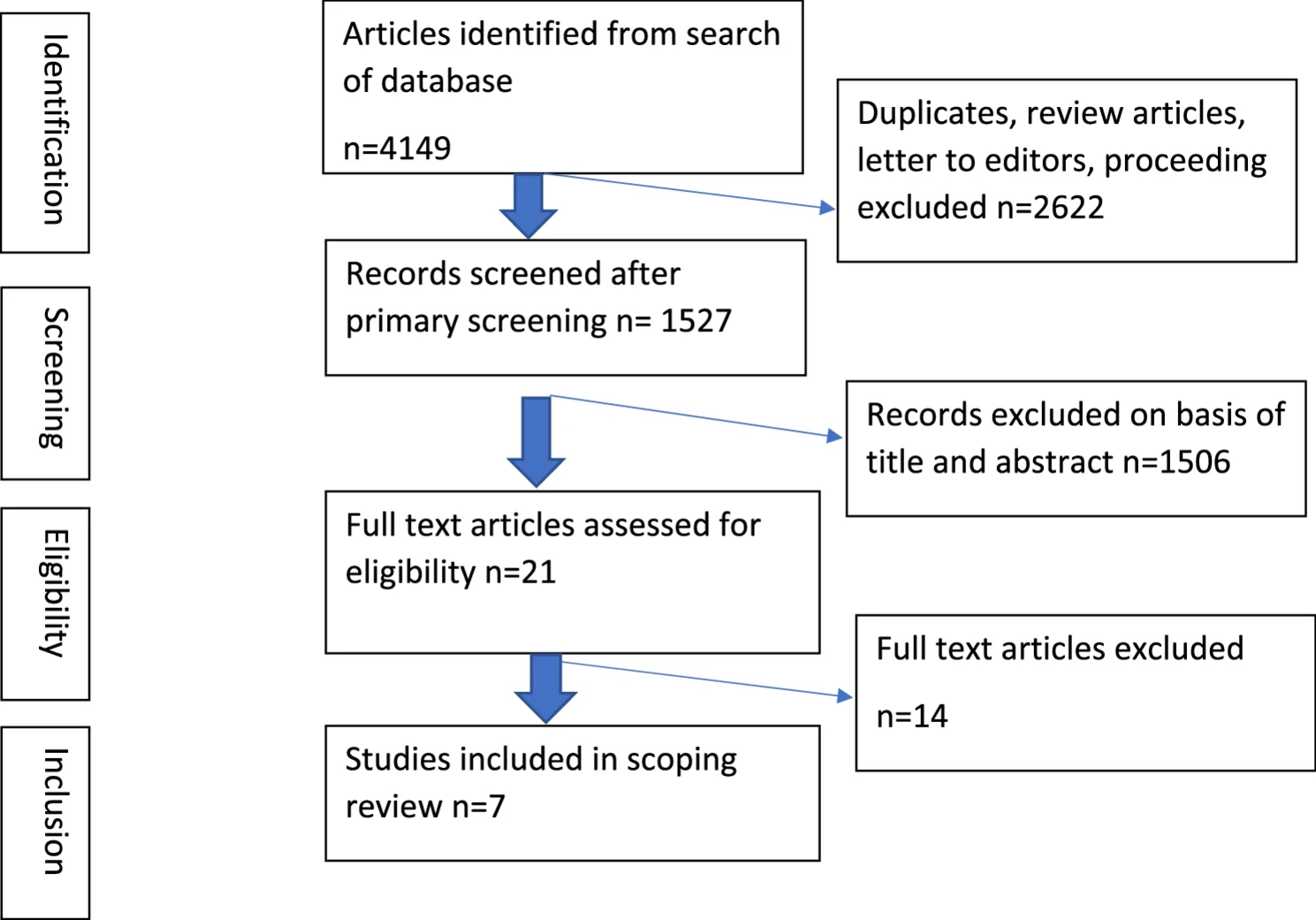
PRISMA flow diagram of the search strategy and selection process

The flow chart in Fig. 1 depicts the research results and selection of eligible studies. The initial search generated a total of 4149 articles. After removing duplications and nonrelevant studies, 21 full-text articles were included for further analysis. Seven of them who met the eligibility criteria were included in the study. The main results of the excluded studies were case reports and series with fewer than five pregnancies. All review articles and conference proceedings were excluded.

Table 1 presents the characteristics of the primary studies, including the year of the study, author, title of the study, country of origin, study time, type of study, and sample size. Tables 2 and 3 present the main information of the included studies. The distribution of the years of the included studies was wide (between 1970 and 2023): two in the 1970s, two in the 1980s, and three between 2010 and 2023.

**Table 1:** The characteristics of the seven included studies

Study year	Journal name	Author	Title	Study place	Study type	Number of pregnant women	Number of pregnancies
1970	Am J Obstet Gynecol.	Tyson JE [1]	Obstetric and gynaecologic considerations of dwarfism	Baltimore, North America	Retrospective	3	7
1970	Ann Hum Genet	Murdoch JL [9]	Achondroplasia–a genetic and statistical survey	Baltimore, North America	Retrospective	27	50
1982	J Reprod Med.	Lattanzi D [5]	Achondroplasia and pregnancy	Pittsburgh, North America	Retrospective	4	5
1986	Obstet Gynecol.	Allanson J [3]	Obstetric and gynaecologic problems in women with chondrodystrophies	Vancouver, North America	Cross sectional	26	47
2016	Can J Anaesth.	Lange EMS [8]	Anesthetic management for Cesarean delivery in parturient with a diagnosis of dwarfism	Chicago, North America	Retrospective	7	8
2023	Genet Med.	Brar BK [10]	Route of delivery does not impact postnatal surgical morbidity in pregnancies affected by fetal achondroplasia	Baltimore, North America	Cohort study	NA	96
2023	Proc (Bayl Univ Med Cent).	Dumitrascu CI [7]	Anesthetic management of parturient with achondroplasia: a case series.	Arizona, North America	Retrospective	7	16

**Table 2:** Antenatal complications of pregnancy with achondroplasia in the included studies

Author	The age at conception (Years) (Mean)(range)	Height (cm) (Mean)(range)	Weight (kg) (Mean)(range)	Abortion/MTP	Anemia/GDM	Hypertensive disease in pregnancy (HIP)	Cardio respiratory/and neurological	Others
Tyson JE [1]	NA	123.9	42.6	1 patient with abortion	2 GDM	NA	NA	9 married to 9 dwarfs
Murdoch JL [9]	26.5	123.44	45	5 -abortions	NA	NA	NA	7 married to average stature, 20 married to achondroplasia
Lattanzi D [5]	24 (18–28)	NA	NA	NA	Iron deficiency anemia	Eclampsia, Preeclampsia, polyhydramnios Supine hypotension	NA	PROM, 3 primi and 2 s para 2 preterm delivery
Allanson J [3]	26.7	NA	NA	NA	NA	NA	NA	Not clearly mentioned
Lange EMS [8]	NA	122.5 (114–135)	NA	NA	NA	NA	NA	One among eight preterm, multiple attempts for spinal, CSF can not be withdrawn, conversion to GA (inadequate neuraxial block), high spinal blockage, 1 minor, 4 difficult placements, 3- no issue
Brar BK [10]	NA	NA	NA	NA	NA	NA	NA	NA
Dumitrascu CI [7]	25.6 (20–34)	126.6 (121–139)	62.4 (47.2–90)	NA	NA	NA	NA	5 primigravida, 5 s gravida, 2 third gravida, 4 MTP,

*NA *Not available, *MTP* Medical termination of pregnancy

**Table 3:** Intrapartum and postpartum complications

Author	Period of gestation Weeks -Mean (range)	Mode of delivery	Anesthesia	Fetal weight (kg)	Live births (number)	Maternal complication	Fetal complication
Tyson JE [1]	36 (35–37)	All CD	NA	2.65	4 with one achondroplasia	2 maternal respiratory embarrassment	2 neonatal deaths
Murdoch JL [9]	NA	Majority CD	NA	2.94 (male) 2.67 (female)	45	NA	AF (8), AM (10), N (12), NND (8), A (6), HC (1)
Lattanzi D [5]	34.5 (28–39)	All CD except one with 750 g	All under GA except one at 28 weeks	2.17 (0.75–3.62)	4 with one achondroplasia	Postpartum paralysis	NND-1, hypospadias, one neonate with achondroplasia Symptoms started at 20 weeks
Allanson J [3]	NA	All CD	Spinal or epidural (12) General	NA	NA	4 – Nerve root compression 4-respiratory difficulty	NA
Lange EMS [8]	37.5 (30.6–39.1)	All CD	Spinal epidural 4, Spinal − 1, epidural 3 2/8 - GA	2.51 kg average (1.57–3.13 kg)	NA	Paraesthesia following spinal	NA
Brar BK [10]	NA	All CD	NA	NA	NA	NA	NA
Dumitrascu CI [7]	38.2 (36–39)	All CD	3 epidurals 4 spinals 5 General anesthesia	NA	NA	Transient paraesthesia − 3 Postanaesthetic fetal deceleration, hysterotomy and vacuum 1	NND − 1, Low Apgar − 2 One re-exploration

*NA* Not available, *CD* Cesarean delivery, *GA* General anesthesia, *NND* Neonatal death, *AF* Achondroplasia female, *AM* Achondroplasia male, *A* Achondroplasia, *HC* Hypochondroplasia, *N* Normal

### Primary studies

The characteristics of the included studies are presented in Table 2. All were retrospective studies or case series. A few included studies did not focus exclusively on pregnant women with achondroplasia; rather, they focused on dwarfism and pregnancies with more than 5 women with achondroplasia in an original article. A total of 229 women with achondroplasia pregnancies were included in seven studies.

#### Pregnancy observations

##### Height and weight

The mean height observed was 122–126 cm in different studies, with a minimum of 114 cm and a maximum of 149 cm. The mean weight was 42.6–62.4 kg.

#####  Age at pregnancy

Most pregnancies were observed in the third decade of life. It was reported in four studies [1, 7–9]. In the era of 1970, the average age of a woman’s first pregnancy was 21 years, and the average parity age was 24 years; at present, it is 27 years in the USA. Four studies revealed that the average age was between 24 and 26.7 years [3, 5, 7, 9].

### Pregnancy loss, including medical termination of pregnancy (MTP)

The incidence of pregnancy loss varied across these studies. The overall rate of pregnancy loss was 10% in the study by Murdoch [9] and 14% by Tyson [1] (pregnancy loss). Dumitrascu [7] reported a 25% rate for MTPs. Many studies (mainly on anesthesia) did not mention the rate of pregnancy loss in their series.

### Antenatal complications

The majority of the included articles did not report consistent antenatal complications such as gestational diabetes mellitus (GDM), hypertension during pregnancy, or anemia. Lattanzi et al. [5] reported a 40% incidence (2 in 5 pregnancies) of blood pressure-related medical and obstetric complications. The incidence of pregnancy-related diabetes and iron deficiency anemia was 1 in 5 pregnancies in their study. They also reported supine hypotension syndrome [5]. Tyson also reported GDM in 2 of 6 pregnancies [1].

Respiratory manifestations are not uncommon symptoms reported in the included articles. Shortness of breath, compensatory tachypnea, cyanosis, respiratory acidosis, and cor pulmonale were the main respiratory symptoms reported in two studies.

### Delivery

Planned delivery between 35 and 37 weeks was reported in the majority of the studies [1], with a range between 28 weeks and 39 weeks. Indicated preterm deliveries were documented in 12.5% − 40% of cases as a result of cardiorespiratory problems or severe preeclampsia [1, 5]. The most recent publication, 2023, mentioned a higher mean gestational age at delivery, i.e., 38.2 weeks of pregnancy [7]. Five out of seven studies reported that all deliveries were conducted by cesarean section due to cephalopelvic disproportion (CPD). Two studies investigated vaginal delivery. Most vaginal deliveries are conducted because of maternal and fetal morbidity [5, 9]. Most vaginal deliveries were noted among preterm babies.

Difficult delivery of the fetal head requiring extension of the incision and instruments during cesarean section was reported by Dumitracsu [7]. The use of vacuum-assisted cesarean delivery and prolonged fundal pressure was necessary. Warning regarding uterine tears was mentioned in the study [7].

### Neonatal morbidity and mortality

Neonatal mortality has been reported to be between 6.3% and 28% in different studies [1, 5, 7, 9]. A total of 11 cases of neonatal mortality were noted in these studies. Most of these neonates (10/11) had features of achondroplasia. The main reasons were respiratory depression and low Apgar scores. The life expectancy of infants born to mothers with achondroplasia is similar to that of infants born to mothers with average stature. If the neonatal period survived, then life expectancy was considered to be more than six decades. The number of neonates born at average for gestational age neonates was greater than the number of neonates who were small for gestational age neonates. Infants born to mothers who have achondroplasia have a 50% chance of having achondroplasia. The frequency of achondroplasia among infants was 50% according to the methods of Murdoch, as prenatal diagnosis was not widely accepted. Maternal polyhydramnios was considered an indirect indicator of poor fetal outcomes in a study by Lattanzi [5]. Twenty-six neonates out of 53 live births were diagnosed with achondroplasia in 3 studies [1, 5, 7].

### Postpartum morbidity and mortality

Cesarean delivery was associated with anesthetic morbidity and airway complications. Postpartum paralysis was reported in 1/229 pregnancies, and other morbidities included nerve root compression and transient paraesthesia. A narrow spinal canal enclosing a relatively normal spinal cord predisposes patients to neurological disorders. Numbness and tingling of the lower limbs were attributed to lumber canal stenosis, which became exaggerated during pregnancy because of lumber lordosis. Hence, prenatal issues such as backache and neurological symptoms are common [11].

### Anesthetic considerations

A significant challenge for anesthetics is selecting and providing anesthesia to women with achondroplasia, as stated in the majority of studies [12–14]. Owing to facial and spinal differences, regional and general anesthesia has its merits and demerits. Small mouth opening, maxillary hypoplasia, a flattened nasal bridge, a short thyromental distance, temporomandibular joint rigidity, pharyngeal narrowing, atlantooccipital instability, and limited neck extension made intubation for general anesthesia challenging. Apart from difficult intubation and rapid desaturation (due to pulmonary alterations and sleep apnea), it was found. The correct dose, depending on height and weight, was followed to prevent complications. Fiber-optic endotracheal intubation and video laryngoscopy were recommended to reduce risks, particularly in patients with evidence of cervicomedullary compression.

Both spinal anesthesia and epidural anesthesia were used [8]. However, altered spinal anatomy and unpredictable drug spread are specific concerns. Difficulty in obtaining the lumbar space due to previous surgery or lumbar stenosis led to access difficulties. Significant hypotension due to the dual effects of neuraxial block and aortocaval compression by the gravid uterus was also noted. Hence, epidural or incremental spinal anesthesia was preferred in few studies. Invasive arterial pressure monitoring was needed for safe outcomes. Recent studies have reported successful neuraxial anesthesia. No general anesthesia conversion was noted [7].

## Discussion

Short stature includes an adult height of less than 146 cm in women. Various pathologies resulting in short stature are skeletal dysplasia, endocrine disturbances, and constitutional and metabolic conditions. Although excellent best-practice guidelines exist for the prenatal and perinatal evaluation of pregnant women with skeletal dysplasia [12], there are no specific guidelines for pregnant women with achondroplasia. Hence, a scoping review addressing pregnant achondroplasia women is conducted.

### Publication trends

Almost all the studies originated from the USA. In the literature search, several review papers, including guidelines, were identified. They are useful; however, most of them are not specified for achondroplasia. Most of the published work consists of case reports and clubbing of various types of skeletal dysplasia. Few articles in the recent era have specifically highlighted anesthetic problems associated with achondroplasia. However, detailed obstetric and neonatal information concerning pregnant women with achondroplasia has not been described effectively. There is little information in the published literature concerning the effective management of pregnant women with achondroplasia.

The majority of information is observational, and the major concern is labor and delivery. A systemic approach involving delivery and anesthesia has been developed for skeletal dysplasia and pregnancy. Although most of the guidelines are relevant to pregnancies in achondroplasia, guidelines specific to achondroplasia have not been formulated.

### Antenatal issues

Achondroplasia with kyphoscoliosis may benefit from preconception pulmonary function tests. More research is needed to address such specific issues. This would identify patients at high risk of cardiopulmonary embarrassment [3]. The antenatal period of a woman with achondroplasia is associated with a similar risk of abortion to that of the general population, recurrence, hypertensive disorders during pregnancy, cardiopulmonary consequences and preterm termination of pregnancy.

Standard guidelines on weight gain during pregnancy are not applicable; however, weight gain of 5–9 kg over the course of pregnancy is considered reasonable.

All the women had achondroplasia with lumbar lordosis and short vertebral pedicles, and many also experienced wedging of the upper lumbar vertebrae and stenosis. However, not every woman can experience respiratory symptoms during pregnancy. Similarly, many average-stature women might have shortness of breath (SOB) from pregnancy. The frequency of respiratory difficulty was not consistently observed among pregnant women with achondroplasia. SOB was more common when achondroplasia was associated with severe kyphoscoliosis, wherein restrictive chest conditions led to reduced total lung capacity and vital capacity. In addition, the short trunk, the reduced distance from the xiphisternum to the symphysis, and the narrow intercristal distance made the uterus abdominal early in pregnancy without optimum usage of the pelvic cavity. This exacerbates respiratory symptoms [1]. It is an important cause of maternal morbidity and mortality. Hence, preterm delivery was needed to relieve respiratory symptoms in such pregnancies.

It is important to identify pregnancies associated with cardiorespiratory embarrassment. This is one of the most important objectives of antenatal care. Careful examination and assessment of the cardiovascular and respiratory systems throughout pregnancy in women with achondroplasia are suggested for early diagnosis and treatment of concern issues. Referral to fetal medicine centers for prenatal diagnosis is crucial. A woman with achondroplasia and associated kyphosis may face pulmonary issues from midgestations, which should be managed by position and pulmonary support.

Preterm delivery may be indicated in such cases after the benefit of maternal respiratory condition to prematurity is weighed. Preferably, delivery should be at tertiary centers. Pregnant women with achondroplasia are not at higher risk of spontaneous preterm labor; however, the treatment of preterm labor does not differ from that of the general population. Fluid overload should be prevented, and infusion should depend upon maternal stature. Almost identical fetal wastage to that of the general population is noted. Greater wastage was noted when it was related to defects in the germplasms of both achondroplasia couples. Newborn achondroplasia might need higher center/specialized care postdelivery; hence, appropriate counseling is necessary.

### Route of delivery

The vaginal and pelvic findings in a woman with achondroplasia are different. It is characterized by a narrow and short vagina approximately 8 cm in length. The iliac blades are flattened, giving a champagne-glass appearance to the pelvic inlet. Narrow and short sacrosciatic notches, blunt ischial spines, short ilia, easy palpation of sacral vertebrae, and broad, flat, and short pelvises at the cavity are typical bony features. Shortening of the anteroposterior diameter of the pelvis precludes normal delivery. A large head size of the fetus in mothers with achondroplasia who are pregnant with babies who have achondroplasia is responsible for a high incidence of malpresentations (breech> other malpresentation) and Cephalo-pelvic disproportion (CPD), leading to a higher probability of cesarean deliveries. In addition, cesarean delivery is used for almost all cases of cephalopelvic abortion. It is preferred by senior consultants. For cosmetic and functional reasons, a lower section (Pfannenstiel incision) is preferred.

### Challenges with anesthesia

A significant challenge for anesthetics is common in patients with achondroplasia [13, 14]. The common issues associated with neuraxial surgery include prolonged placement time, technically challenging procedures, inadequate neuraxial anesthesia, unilateral blockage, unintentional dural puncture, the use of an intrathecal catheter, and total spinal blockage. Skilled anesthetics are essential for the management of airway difficulties. Hence, the results of several studies showing no difficult intubation cannot be generalized to the safety of tracheal intubation in pregnant patients with achondroplasia. Hence, advanced airway equipment should be kept ready.

Modification of the plan for anesthesia is necessary for safety. Patient-specific risk should be evaluated appropriately through medical history and preparation, including spinal imaging, arterial blood gas studies, and cardiac/pulmonary evaluations. Neuraxial ultrasound can be considered. Advanced planning for anesthesia is suggested whenever possible. An increased risk of general and spinal anesthesia should be conveyed to the couple.

### Social concerns

In the ideal world, people with dwarfism must not face social stigma and family/societal rejection. Some organizations recruit and educate only dwarfs, their families, and the public with the sole aims of social participation, mate selection, and psychological advice. They are average in intelligence. The social detriment of patients with dwarfism can reduce social and marital opportunities. Contraceptive advice is essential and similar to that of any other postpartum woman.

### Strengths

This is the first scoping review of a rare combination of pregnancy and achondroplasia. This is the only study that combined a description of ante-natal, perinatal, and postnatal anesthetic complications in pregnant women with achondroplasia. An attempt was made to extract all available data on pregnancy and related complications. This study could be useful for formulating obstetric and anesthesia guidelines specific to pregnant women with achondroplasia.

### Limitations

This study has several inherent limitations associated with the inclusion of retrospective studies, including possible incomplete or missing data in the selected studies and uncertainty in the reason for managing decisions. Given that all included studies were from North America, the global perspective might be missing from this scoping review.

Although the included case series studies described the anesthetic management of parturients with achondroplasia, this study cannot provide definite recommendations. Moreover, this review spans 22 years, during which time the changes in anesthetic and obstetric practices invariably evolved. Further research, including large-scale, prospective studies, is needed to provide a greater level of evidence for safe as well as effective anesthesia and delivery of parturients with achondroplasia.

### Implications and future directions

This study provides baseline information on the obstetric and anesthetic implications and complications in pregnant women with achondroplasia. This article adds important information on hypertension, eclampsia, abortion, medical termination of pregnancy, etc., to the existing guidelines for pregnant women with skeletal dysplasia.

Over the past few decades, the number of global publications on achondroplasia has increased, but the number of articles concerning pregnant women with achondroplasia has remained low. In the era of genetics, the incidence of familial cases of achondroplasia is decreasing because of the availability and detection of genetic mutations and prenatal diagnosis [15, 16]. However, sporadic cases of achondroplasia may be the same in the future. Owing to the equal distributions of both genders and the good life expectancy of achondroplasia patients, pregnancy in women with achondroplasia may not be an uncommon scenario in the future. Hence, multicentric studies specifically addressing pregnant women with achondroplasia should be initiated. Data specific to antenatal, perinatal and postnatal obstacles and complications related to obstetrics and anesthesia should be collected to form formal guidelines specific to pregnancies in women with achondroplasia.

## Conclusion

A significant pregnancy-related complication was noted in a woman with achondroplasia. Multicenter studies should be initiated in accordance with methodological reference standards to strengthen the reliability and generalizability of the findings and to increase their relevance for implementation in clinical practice.

## Data Availability

No datasets were generated or analysed during the current study.

## References

[1] Tyson JE, Barnes AC, McKusick VA, Scott CI, Jones GS. Obstetric and gynecologic considerations of dwarfism. Am J Obstet Gynecol. 1970;108(5):688–704. 4990504 10.1016/0002-9378(70)90534-x

[2] Pauli RM. Achondroplasia: a comprehensive clinical review. Orphanet J Rare Dis. 2019;14(1):1. 30606190 10.1186/s13023-018-0972-6 PMC6318916

[3] Allanson JE, Hall JG. Obstetric and gynecologic problems in women with chondrodystrophies. Obstet Gynecol. 1986;67(1):74–8. 3940342

[4] Carney MH, Holt L, Kumar A, Duncanson L, Woodard L, Mckeon BA. Barriers to Obstetric and Gynecologic Care for Women Who Use Wheelchairs [10B]. Obstet Gynecol. 2020;135:20S.

[5] Lattanzi DR, Harger JH. Achondroplasia and pregnancy. J Reprod Med. 1982;27(6):363–6. 6889650

[6] Fredwall SO, Maanum G, Johansen H, Snekkevik H, Savarirayan R, Lidal IB. Current knowledge of medical complications in adults with achondroplasia: A scoping review. Clin Genet. 2020;97(1):179–97. 30916780 10.1111/cge.13542 PMC6972520

[7] Dumitrascu CI, Eneh PN, Keim AA, Kraus MB, Sharpe EE. Anesthetic management of parturients with achondroplasia: a case series. Proc (Bayl Univ Med Cent). 2024;37(1):63–8. 38173994 10.1080/08998280.2023.2261084 PMC10761160

[8] Lange EM, Toledo P, Stariha J, Nixon HC. Anesthetic management for Cesarean delivery in parturients with a diagnosis of dwarfism. Can J Anaesth. 2016;63(8):945–51. 27174298 10.1007/s12630-016-0671-5

[9] Murdoch JL, Walker BA, Hall JG, Abbey H, Smith KK, McKusick VA. Achondroplasia–a genetic and statistical survey. Ann Hum Genet. 1970;33(3):227–44. 5504223 10.1111/j.1469-1809.1970.tb01648.x

[10] Brar BK, Bober MB, Gough E, Hashmi SS, Hecht JT, Dujmusic L, et al. Route of delivery does not impact postnatal surgical morbidity in pregnancies affected by fetal achondroplasia. Genet Med. 2023;25(7):100845. 37061874 10.1016/j.gim.2023.100845 PMC12131108

[11] Ain MC, Abdullah MA, Ting BL, Skolasky RL, Carlisle ES, Schkrohowsky JG, et al. Progression of low back and lower extremity pain in a cohort of patients with achondroplasia. J Neurosurg Spine. 2010;13(3):335–40. 20809726 10.3171/2010.3.SPINE09629

[12] Savarirayan R, Rossiter JP, Hoover-Fong JE, Irving M, Bompadre V, Goldberg MJ, et al. Best practice guidelines regarding prenatal evaluation and delivery of patients with skeletal dysplasia. Am J Obstet Gynecol. 2018;219(6):545–62. 30048634 10.1016/j.ajog.2018.07.017

[13] Cohen SE. Anesthesia for Cesarean section in achondroplastic dwarfs. Anesthesiology. 1980;52(3):264–6. 7369515 10.1097/00000542-198003000-00014

[14] Brown A, Liu H, Chandler C. Anesthetic dilemmas in an achondroplastic patient undergoing elective cesarean section. J Biomed Res. 2024;38(5):1–4. 10.7555/JBR.37.20230301 PMC11461534 38832540

[15] Vivanti AJ, Costa JM, Rosefort A, Kleinfinger P, Lohmann L, Cordier AG, et al. Optimal noninvasive diagnosis of fetal achondroplasia combining ultrasonography with circulating cell-free fetal DNA analysis. Ultrasound Obstet Gynecol. 2019;53(1):87–94. 29380944 10.1002/uog.19018

[16] Chitty LS, Mason S, Barrett AN, McKay F, Lench N, Daley R, et al. Noninvasive prenatal diagnosis of achondroplasia and thanatophoric dysplasia: next-generation sequencing allows for a safer, more accurate, and comprehensive approach. Prenat Diagn. 2015;35(7):656–62. 25728633 10.1002/pd.4583 PMC4657458

